# Optical Coherence Tomography Angiography Is Associated with Disease Activity Expressed by NEDA-3 Status in Patients with Relapsing Multiple Sclerosis

**DOI:** 10.3390/jcm14207370

**Published:** 2025-10-18

**Authors:** Jozef Szilasi, Marianna Vitková, Zuzana Gdovinová, Miriam Fedičová, Pavol Mikula, Lýdia Frigová, Jarmila Szilasiová

**Affiliations:** 1 Department of Ophthalmology, Faculty of Medicine, Pavol Jozef Šafárik University, Louis Pasteur University Hospital, 04011 Košice, Slovakia; jozef.szilasi@upjs.sk; 2 Department of Neurology, Faculty of Medicine, Pavol Jozef Šafárik University, Louis Pasteur University Hospital, 04011 Košice, Slovakia; marianna.vitkova@upjs.sk (M.V.); zuzana.gdovinova@upjs.sk (Z.G.); miriam.fedicova@unlp.sk (M.F.); 3 Department of Social and Behavioral Medicine, Pavol Jozef Šafárik University, 04011 Košice, Slovakia; pavol.mikula@upjs.sk; 4 ProMagnet, Magnetic Resonance Imaging, 04001 Košice, Slovakia; lydia.frigova@promagnetkosice.sk

**Keywords:** multiple sclerosis, disease activity, optical coherence tomography angiography, NEDA-3

## Abstract

**Background:** Retinal microvascular changes may serve as biomarkers for disease activity in multiple sclerosis (MS). This study evaluated macular and peripapillary vascular plexus densities using optical coherence tomography angiography (OCTA) in patients with relapsing MS (RMS) and healthy controls (HCs), exploring their association with disease activity based on the NEDA-3 concept. **Methods:** In a cross-sectional study, 117 RMS patients and 37 HCs underwent OCTA imaging. Parameters analyzed included superficial vascular plexus (SVP), deep vascular plexus (DVP), foveal avascular zone (FAZ), and radial peripapillary capillary (RPC) density. Images with artifacts were excluded. Associations between OCTA metrics and demographic, clinical, and MRI volumetrics, as well as NEDA-3 status, were evaluated using multivariate generalized estimating equations. Receiver operating characteristic (ROC) curves assessed predictive capacity. **Results:** Compared to HCs, MS eyes with prior optic neuritis showed significantly lower SVP density (*p* < 0.05). DVP and FAZ parameters did not differ between groups. SVP and DVP densities correlated with age, disease duration, relapse history, and MRI volumetrics, including gray matter and whole brain volume. SVP density predicted NEDA-3 status (AUC = 0.82), while DVP also showed predictive value (AUC = 0.64). FAZ FD (Foveal density) was associated with gray matter and whole brain atrophy (AUC = 0.62–0.61). **Conclusions:** Retinal vascular alterations correlate with clinical and MRI measures in MS. Reduced SVP and DVP densities may serve as markers of recent disease activity, and FAZ metrics reflect neurodegeneration. OCTA may be a valuable non-invasive tool for monitoring MS progression.

## 1. Introduction

Multiple sclerosis (MS) is an inflammatory, demyelinating disease of the central nervous system (CNS) characterized by an unpredictable clinical course. Neuro-axonal loss, including damage to the optic nerves, underpins chronic neurodegeneration and contributes to disability [1]. CNS inflammation can induce hypoxia and hypoperfusion by damaging mitochondria and endothelial cells, with accumulating evidence supporting a “hypoxia–inflammation cycle” that exacerbates energy failure and promotes disease progression [2,3].

Several studies have documented reduced cerebral blood flow in MS patients, even prior to the appearance of structural damage, suggesting hypoperfusion occurs early in the disease course [4,5,6]. Exploring retinal vascular alterations offers a non-invasive window into microvascular changes and the role of hypoperfusion in MS pathophysiology [2]. Optical coherence tomography angiography (OCTA) is a novel, non-invasive imaging modality that visualizes retinal and choroidal vasculature with high resolution [7].

Research indicates that retinal microcirculation blood flow is diminished in MS patients compared to healthy controls, with reductions observed even in eyes unaffected by optic neuritis [8,9,10,11]. Notably, OCTA-derived metrics correlate with retinal layer thickness, disability scores, and disease activity [10,12,13]. Specifically, vessel densities in the superficial vascular plexus (SVP) and deep vascular plexus (DVP) are decreased in MS and correlate inversely with disease duration. Lower macular SVP vessel density has been linked to higher Expanded Disability Status Scale (EDSS) scores and poorer visual acuity [2].

This study aims to compare retinal vascular densities in a large cohort of people with MS (PwMS) to healthy controls (HCs) and assess whether OCTA parameters associate with disease activity, as defined by NEDA-3 status (no relapses, disability progression, or MRI activity), including brain volume metrics. We also investigate correlations between OCTA findings and demographic, clinical, and MRI volumetric variables, utilizing the Icobrain software. To our knowledge, these parameters have not been extensively studied together in this context, and our findings may provide insights into the microvascular contributions to MS disease activity.

## 2. Materials and Methods

### 2.1. Study Design

This observational, cross-sectional study was conducted at the Neurology and Ophthalmology Departments of Pavol Jozef Šafárik University Medical Faculty and Louis Pasteur University Hospital in Košice, Slovakia, from January 2024 to September 2025.

### 2.2. Study Population

**Figure 1:**
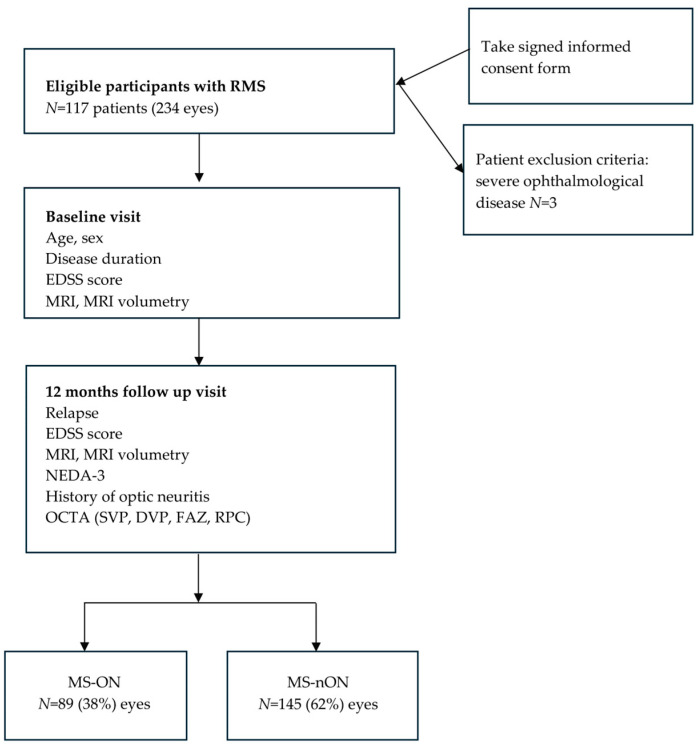
A schematic flowchart diagram showing the procedure by which data were tested in RMS patients.

A total of 117 consecutive patients with relapsing multiple sclerosis (RMS) were recruited based on inclusion and exclusion criteria (Figure 1). Inclusion criteria comprised (1) diagnosis of MS according to the 2017 McDonald criteria [14], (2) age >18 years, and (3) ability to provide written informed consent. Exclusion criteria included (a) retinal pathology per OSCAR-IB criteria [15], (b) prior ocular surgery or trauma, (c) recent optic neuritis (ON) within 6 months, (d) uncontrolled hypertension or diabetes mellitus, (e) refractive errors >6 diopters, (f) recent MS relapse or corticosteroid treatment within 6 months, (g) other CNS or autoimmune disorders, and (h) pregnancy. Three patients were excluded: one with cataract and two with retinal dystrophy.

Healthy controls (HCs) were volunteers without autoimmune or ocular diseases, recruited mainly from hospital staff. The final cohort consisted of 117 RMS patients (234 eyes) and 37 HCs (74 eyes). All procedures conformed to ethical standards.

### 2.3. Study Procedures

Participants underwent two visits over 12 months: baseline and follow-up (FUV). Clinical assessments included neurological examination with EDSS scoring [16], ophthalmological evaluation, OCTA imaging, and brain MRI. Disease duration was calculated from initial MS symptom onset to OCTA examination. Disease activity was assessed by NEDA-3 status (see Section 2.3.3). Medical and MRI data were retrieved from records. ON diagnosis followed the criteria from the Optic Neuritis Treatment Trial [17], confirmed by MRI, OCT, or visual evoked potentials.

All patients received disease-modifying therapy (DMT) according to national guidelines. MRI scans were performed within 3 months of the OCTA assessment and evaluated blindly by radiologists.

#### 2.3.1. Optical Coherence Tomography Angiography (OCTA)

OCTA imaging was performed at FUV using the Optovue Solix device (Optovue, Inc., Solix 2019 V1.0.0.382, Fremont, CA, USA). Scans covered 6 mm × 6 mm areas of both eyes, acquired under low-light conditions without pupillary dilation by an experienced ophthalmologist. The device employs an 840 nm wavelength, capturing 120,000 A-scans per second with 5 µm axial resolution, and utilizes DualTrac Motion Correction for minimizing motion artifacts. Blood flow was visualized using the device’s AngioAnalytics (Angiovue software SOLIX FullRange AngioVue Expert) based on the SSADA algorithm. OCTA image quality was assessed using the device Q-value, which integrates signal intensity, motion, and focus parameters; scans with Q-values below the threshold corresponding to approximately 25 dB were excluded. Automated segmentation distinguished the superficial vascular plexus (SVP) and deep vascular plexus (DVP).

In addition to macular scans, a 4.5 mm × 4.5 mm peripapillary scan centered on the optic nerve head was acquired using the Radial Peripapillary Capillary (RPC) protocol, which evaluates the microvascular network surrounding the optic disc, extending from the internal limiting membrane to the retinal nerve fiber layer. This protocol and its parameters are well established for the Optovue/AngioVue platform [13].

The OCTA parameters analyzed in this study were automatically generated by the AngioAnalytics software as follows:

- SVP (Superficial Vascular Plexus): vessel density in the superficial retinal layer extending from the internal limiting membrane to the inner plexiform layer (in %).
- DVP (Deep Vascular Plexus): vessel density in the deep retinal layer spanning the inner nuclear layer to the outer plexiform layer (in %).
- FAZ area: area of the foveal avascular zone measured in mm^2^.
- FAZ perimeter: length of the boundary surrounding the FAZ in mm.
- FAZ FD (Foveal Density): represents the vessel density within a 300-μm radius around the foveal avascular zone (FAZ), as automatically generated by the SOLIX software (version Solix 2019 V1.0.0.382) (FD-300) (in %).
- RPC (Radial Peripapillary Capillary) density: vessel density in the peripapillary region surrounding the optic nerve head (in %).

All images underwent masked qualitative review for artifacts by a single rater (J.S.). Out of a total of 234 eyes, approximately 12 images (~5%) were excluded due to significant artifacts, including motion, segmentation errors, or media opacity. This low exclusion rate reflects the use of modern OCTA technology, rigorous quality control, and selective inclusion/exclusion criteria that minimize ocular and systemic confounders, such as uncontrolled hypertension, diabetes mellitus, significant refractive errors, or cataract.

#### 2.3.2. Brain MRI Protocol and Analysis

MRI was performed on a Philips Ingenia 3.0T Omega HP (Philips Medical Systems, Best, The Netherlands) scanner using standardized protocols: 3D T1-weighted MPRAGE and 3D FLAIR sequences. Lesion analysis included T2, FLAIR, T1, and Gd-enhanced sequences, with longitudinal co-registration for lesion tracking. Brain volumetric measures—whole brain (WB), gray matter (GM), and annual brain volume loss (BVL)—were obtained using Icobrain MR software (icometrix NV, version 5.13.1, Leuven, Belgium) [18]. WB and GM volumes were normalized for skull size, with volumes below the 5th percentile indicating atrophy [19,20].

#### 2.3.3. NEDA-3 Assessment

NEDA-3 status was evaluated at FUV based on clinical and MRI data over the previous 12 months. It was defined as the absence of relapses, EDSS worsening, or MRI activity (new/enlarged T2 lesions or Gd-enhancing lesions). EDSS worsening criteria were: ≥1.5 points increase if baseline EDSS = 0; ≥1.0 point if baseline ≤5.0; ≥0.5 points if baseline ≥5.5, sustained for 6 months [21,22]. MRI activity was confirmed by the presence of ≥2 new or enlarged T2 lesions or new Gd-enhancing lesions compared to baseline.

### 2.4. Statistical Analysis

Continuous variables are presented as mean ± standard deviation (SD) or median with interquartile range, categorical variables as counts and percentages. Group comparisons (MS vs. HC, MS-ON vs. MS-nON) were performed with *t*-tests or Mann–Whitney U tests as appropriate. Pearson’s correlation coefficient assessed relationships between variables. ROC curve analysis evaluated the predictive value of OCTA parameters for NEDA-3 status, with the area under the curve (AUC) and 95% confidence intervals calculated. Optimal cut-off points were identified based on Youden’s index. Statistical significance was set at *p* < 0.05. All analyses were conducted using IBM SPSS version 26.0.

## 3. Results

### 3.1. Demographic and Clinical Characteristics

The final cohort comprised 117 patients (234 eyes), of which 89 eyes (38%) had a history of optic neuritis (MS-ON), and 145 eyes (62%) had no prior optic neuritis (MS-ON) (Figure 1).

The demographic, clinical, OCTA, and MRI data of the sample, including patients with relapsing-remitting MS (RMS) and healthy controls (HCs), are summarized in Table 1. The mean age of patients was 38 ± 10.4 years, while that of HCs was 38.9 ± 12.9 years. The median disease duration was 8 years (interquartile range [IQR]: 4–13), with a median EDSS score of 2.5 (IQR: 2–3.5). Seventy-four patients (63%) were female.

Regarding disease-modifying therapies (DMTs), the distribution was as follows: dimethyl fumarate (*n* = 32, 27%), interferons (*n* = 5, 4%), glatiramer acetate (*n* = 5, 4%), natalizumab (*n* = 21, 18%), ocrelizumab/ofatumumab (*n* = 16, 14%), teriflunomide (*n* = 14, 12%), fingolimod (*n* = 1, 1%), cladribine (*n* = 13, 11%), and none in 10 patients (9%). Comparison of demographic characteristics between RMS patients and HCs revealed no significant differences in age or sex (Table 1).

### 3.2. Retinal Vascular Findings

The superficial vascular plexus (SVP) vessel density was significantly lower in MS-ON eyes compared to HCs (48.8 ± 1.68% vs. 49.1 ± 1.28%; *p* < 0.05). Similarly, SVP vessel density was reduced in MS-ON eyes compared to MS-nON eyes (48.8 ± 1.68% vs. 49.3 ± 1.5%; *p* < 0.05). The radial peripapillary capillary (RPC) vessel density was notably decreased in MS-ON eyes relative to HCs (45.4 ± 4% vs. 49.5 ± 2.4%; *p* < 0.001), and also lower compared to MS-nON eyes (45.4 ± 4% vs. 48.5 ± 2.6%; *p* < 0.001). No significant differences were observed in deep vascular plexus (DVP) density or foveal avascular zone (FAZ) parameters between groups.

### 3.3. Correlations

In the entire RMS cohort (*n* = 117), several significant correlations emerged:

- SVP vessel density negatively correlated with age (r = −0.16, *p* < 0.01), time since last relapse (r = −0.25, *p* < 0.001), and disease duration (r = −0.19, *p* < 0.001).
- SVP positively correlated with new FLAIR lesion volume (r = 0.26, *p* < 0.001), whole brain (WB) volume (r = 0.19, *p* < 0.05), and gray matter (GM) volume (r = 0.19, *p* < 0.05).
- DVP vessel density negatively correlated with time since last relapse (r = −0.18, *p* < 0.05), and positively with new FLAIR lesion volume (r = 0.23, *p* < 0.05), WB volume (r = 0.16, *p* < 0.05), and GM volume (r = 0.17, *p* < 0.05).
- FAZ area showed a positive correlation with EDSS score (r = 0.14, *p* < 0.05) and T1 lesion volume (r = 0.19, *p* < 0.05).
- FAZ FD was negatively correlated with age (r = −0.21, *p* < 0.001), time since last relapse (r = −0.21, *p* < 0.001), and disease duration (r = −0.14, *p* < 0.05).
- RPC vessel density negatively correlated with time since last relapse (r = −0.19, *p* < 0.001), disease duration (r = −0.22, *p* < 0.001), and FLAIR lesion volume (r = −0.22, *p* < 0.001). It was positively associated with WB volume (r = 0.27, *p* < 0.001) and GM volume (r = 0.16, *p* < 0.05) (Table 2).

### 3.4. Predictive Analyses

**Figure 2:**
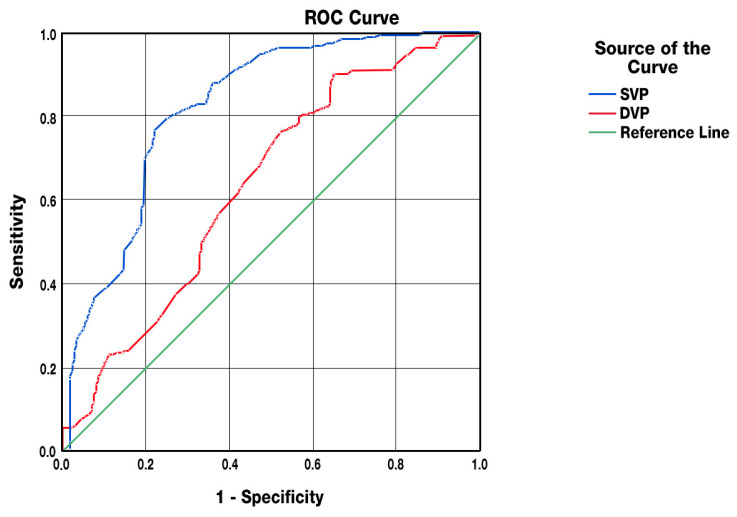
ROC curve of the SVP, DVP, and NEDA-3 status.

**Figure 3:**
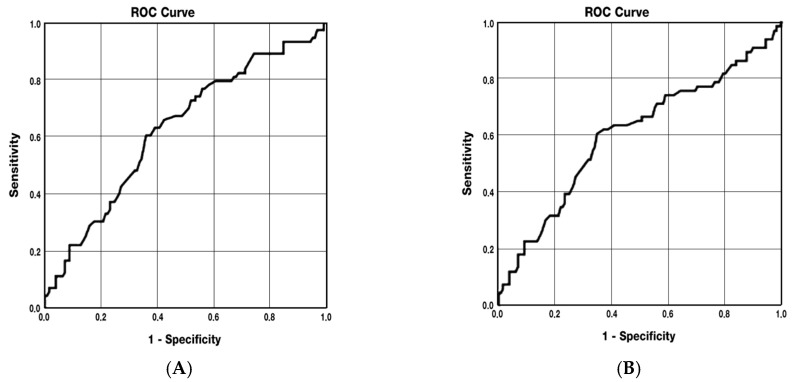
(**A**) ROC curve of the FAZ FD and GM atrophy; (**B**) ROC curve of the FAZ FD and WB atrophy.

Receiver Operating Characteristic (ROC) curve analysis demonstrated that SVP vessel density in RMS patients was significantly associated with NEDA-3 status (AUC = 0.822; 95% CI: 0.767–0.877; *p* < 0.001). The sensitivity and specificity at the optimal cut-off (49.45%) were 77.8% and 78.8%, respectively. Higher SVP values predicted a greater likelihood of achieving NEDA-3. Similarly, DVP vessel density showed an association with NEDA-3 status (AUC = 0.635; 95% CI: 0.562–0.707; *p* < 0.001), with sensitivity and specificity of 76.9% and 50.4%, respectively, at a cut-off of 53.85%. Regarding retinal structural parameters, FAZ FD was predictive of gray matter atrophy (AUC = 0.624; 95% CI: 0.544–0.705; *p* < 0.01), with sensitivity of 63% and specificity of 60% at a cut-off of 51.75%. Higher FAZ FD values were associated with increased risk of GM atrophy. Similarly, FAZ FD correlated with whole-brain atrophy (AUC = 0.606; 95% CI: 0.519–0.693; *p* < 0.05), with a cut-off of 52%, and higher values indicated a higher probability of atrophy development (Figure 2, Figure 3A and Figure 3B). Parameters with an AUC value below 0.7 were considered weaker predictors.

**Figure 4:**
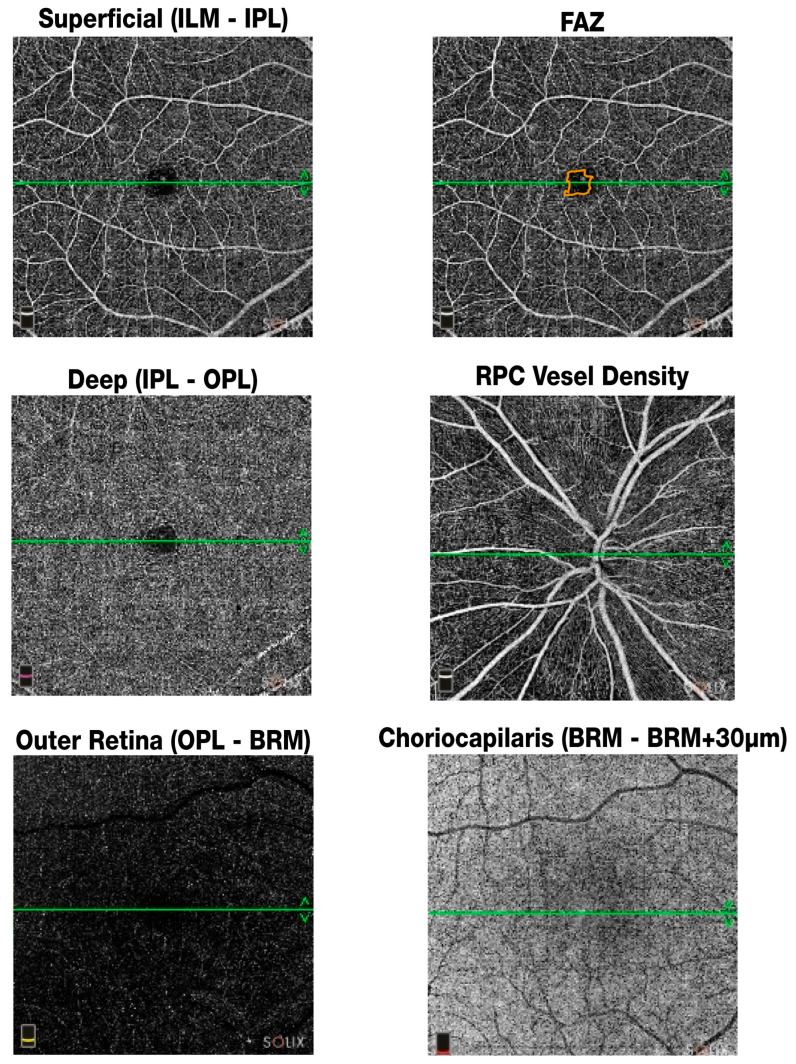
OCTA metrics. Superficial vessel density (VD; ILM–IPL) was measured from the Internal Limiting Membrane (ILM) to the Inner Plexiform Layer (IPL), deep VD (IPL–OPL) from the IPL to the Outer Plexiform Layer (OPL), the Foveal Avascular Zone (FAZ) corresponds to the central macular region, radial peripapillary capillary (RPC) VD was assessed around the optic nerve head, outer retina VD (OPL–BRM) from the OPL to Bruch’s Membrane (BRM), and choriocapillaris VD (BRM–BRM + 30 µm) from Bruch’s Membrane extending approximately 30 µm below it.

Figure 4 illustrates the OCTA metrics assessed in this study using the Optovue Solix device.

## 4. Discussion

### 4.1. Main Findings and Comparison with Previous Studies

In this study, we explored the relationship between retinal vascular densities measured by OCTA and disease activity in a large cohort of people with multiple sclerosis. Our primary aim was to assess whether OCTA-derived parameters are associated with disease activity, as expressed by NEDA-3 status, as well as brain volume metrics.

Optical coherence tomography angiography (OCTA) is a novel, non-invasive imaging modality that enables rapid visualization of the retinal vasculature [23]. Several prior studies have demonstrated reduced retinal vascular measures in patients with S compared to healthy controls (HCs), with associations to visual function and disability scores [10,13,24,25]. Our findings corroborate these observations, showing significant reductions in superficial vascular plexus (SVP) and radial peripapillary capillary (RPC) vessel densities (VD) in MS patients relative to controls. Similarly to previous reports [25,26], we observed decreased VD around the optic nerve head (ONH) in MS eyes, particularly in those with a history of optic neuritis (ON). Interestingly, the deep vascular plexus (DVP) density did not differ significantly between groups, nor did the FAZ parameters, aligning with Murphy et al., who reported unaffected DVP despite reductions in SVP vessel density [10]. However, not all studies have replicated the finding of reduced macular SVP VD in MS, indicating variability that may stem from differences in methodology or cohort characteristics [25].

Notably, the proportion of OCTA images excluded due to artifacts in our study was relatively low (~5%, corresponding to approximately 12 eyes out of 234). This likely reflects the use of the modern Optovue Solix system with DualTrac motion correction, strict signal quality criteria (≥25 dB), careful patient selection, and review by an experienced rater. Only patients without severe refractive errors, cataract, or uncontrolled systemic diseases were included, minimizing potential confounders. Compared to earlier studies such as Murphy et al. (2019), which reported higher artifact rates, our lower rejection rate may be attributed to technological advances in OCTA, optimized image acquisition protocols, and stringent inclusion/exclusion criteria [10]. The selected cohort had minimal comorbidities, with only six patients having arterial hypertension and one patient with type 1 diabetes, further reducing the likelihood of vascular artifacts.

We note that OCTA scans were acquired using 6 × 6 mm scan areas for both eyes, which is preferable for assessment of the inner retina, including the macular RNFL that correlates with the superficial capillary plexus (SCP). This scan size ensures adequate macular coverage while maintaining high image quality and minimizing artifacts.

Additionally, the OCTA parameters analyzed in this study—including SVP, DVP, FAZ area, FAZ FD (foveal avascular zone floor density), and RPC density—were automatically calculated by the Optovue Solix AngioAnalytics software (SOLIX FullRange AngioVue Expert). This ensures standardized quantification of retinal vascular densities and the foveal avascular zone, minimizing operator-dependent variability and facilitating reproducible assessments across all participants. FAZ FD, in particular, represents the vessel density in the periphery of the FAZ and provides additional insight into microvascular integrity in the foveal region.

OCT provides a simple, non-invasive method to monitor retinal structure in PwMS, especially in those with a history of optic neuritis. While OCTA offers additional information on retinal microvasculature, current longitudinal data and evidence on the influence of systemic vascular comorbidities remain limited. At our center, combined OCT and OCTA imaging requires approximately 5–15 min for acquisition, plus ~10 min for interpretation. Correlations between OCTA parameters and other electrophysiological measures such as VEP, as well as structural OCT metrics, were not analyzed in this study but are planned in future investigations to further elucidate relationships between microvascular, structural, and functional retinal changes in MS. Future studies integrating these modalities could enhance understanding of visual pathway integrity and its association with disease activity.

### 4.2. Vascular Changes in Patients with Optic Neuritis

Our data further reveal that MS eyes with a history of ON exhibit lower SVP and RPC VD compared to MS eyes without prior ON, consistent with other studies emphasizing ON’s impact on OCTA measures [10,12,24,27]. This likely reflects reduced metabolic demand in affected optic nerve layers, supported by the strong correlation between SVP density and ganglion cell layer thickness in MS eyes with previous ON. While some studies have reported decreased (peri-)foveal DVP vessel density in MSON compared to MSNON eyes [13,24,28], our findings did not support this, suggesting that ON-related alterations predominantly affect the superficial vasculature. These differences highlight the complexity of vascular involvement and suggest that ON may primarily impact superficial retinal layers, with deeper layers being less affected or affected differently.

### 4.3. Associations Between Vascular Parameters and Neurodegeneration

Across all MS eyes, regardless of ON history, we found that lower SVP and DVP densities correlated with older age, longer disease duration, and extended periods since last relapse. Conversely, higher vessel densities were associated with larger whole-brain (WB) and gray matter (GM) volumes on MRI, aligning with prior observations linking retinal microvascular integrity with neurodegeneration [29]. These associations suggest that retinal vascular alterations may reflect cumulative disease burden and neurodegenerative processes in MS. The positive correlation between vessel densities and brain volumes supports the hypothesis that microvascular health in the retina could serve as a proxy for cerebral neurodegeneration, emphasizing the potential of OCTA in monitoring disability worsening.

### 4.4. Retinal Vascular Parameters as Markers of Disease Activity

An intriguing and novel aspect of our findings is the positive correlation between higher SVP and DVP densities and the volume of new FLAIR lesions, possibly indicating compensatory hyperperfusion during inflammatory activity. This aligns with studies demonstrating increased macular choriocapillaris vessel density during periods of active inflammation [13], implying that retinal vasculature may reflect both current and past disease activity—serving as a surrogate marker for inflammatory and neurodegenerative processes. Moreover, our ROC analysis demonstrated that OCTA parameters could predict NEDA-3 status, with vessel densities above certain thresholds conferring a higher probability of disease stability over the past year. To our knowledge, this is the first study to link retinal vascular metrics with both NEDA-3 and MRI volumetric measures, underscoring OCTA’s potential as a biomarker for disease activity and neurodegeneration in MS.

### 4.5. The Significance of Foveal Avascular Zone (FAZ)

Among the OCTA parameters examined, only FAZ area correlated significantly with disability (EDSS score) and T1-lesion volume, suggesting that the FAZ may be a marker of irreversible neurodegeneration. Larger FAZ areas were associated with higher disability scores, supporting prior reports linking reduced retinal vessel densities with increased disability [10,27]. Lanzillo et al. observed that decreasing parafoveal vessel densities predicted worsening EDSS over a year [30], indicating that the FAZ may serve as an imaging biomarker for disease progression. The enlargement of FAZ could reflect microvascular rarefaction or capillary dropout, leading to tissue ischemia and subsequent neurodegeneration, making it a promising candidate for future longitudinal studies.

### 4.6. Retinal Vascular Changes as a Reflection of Cerebral Neurodegeneration

The positive associations between RPC vessel density and brain volumes reinforce the notion that retinal microvasculature reflects cerebral neurodegeneration. Our findings suggest that microvascular deterioration in the retina parallels atrophic changes in the brain, possibly driven by shared pathogenic mechanisms such as inflammation, microglial activation, or endothelial injury. These relationships highlight the potential utility of retinal vasculature assessments as non-invasive biomarkers for cerebral pathology, offering a window into the ongoing neurodegenerative processes in MS.

### 4.7. Potential Pathophysiological Mechanisms and Implications

The mechanisms underlying retinal vascular changes in MS are not fully understood. Reduced SVP vessel density may result from endothelial injury secondary to prior inflammation, or from decreased metabolic demand due to ganglion cell loss, leading to secondary vascular remodeling. Alternatively, vascular aberrations could be contributory, rather than purely consequential, in MS pathogenesis. Since OCTA captures perfusion patterns but not vessel morphology or integrity, further studies employing advanced imaging and histopathological correlation are needed. Notably, previous work indicates that superficial vessel rarefaction following acute ON evolves into retinal ganglion cell atrophy [31], but such changes are less likely to explain ON-independent vascular alterations, given that DVP is supplied by vessels not directly feeding ganglion cells. We hypothesize that multiple pathological mechanisms—such as glial activation, astrocyte and microglial involvement near vessels—may influence microvascular changes independently of ON [32]. These mechanisms might underlie the associations we observed between retinal vascular densities and brain atrophy, highlighting OCTA’s potential for monitoring neurodegeneration beyond visual pathways.

Published data have demonstrated that systemic vascular factors may modulate retinal microcirculation. Hypertension and diabetes mellitus are known to exert measurable effects on retinal microvasculature, with hypertension altering retinal and choroidal vascular structures detectable by OCTA [33], and diabetes reducing retinal vascular density and affecting FAZ parameters [34]. These findings highlight the importance of considering systemic health status when interpreting OCTA results. We recognize this as an important direction for future research and plan to systematically evaluate the impact of systemic comorbidities such as hypertension and diabetes on OCTA parameters in PwMS to better delineate their potential confounding effects.

OCTA parameters may differ across imaging platforms due to variations in scan protocols and segmentation algorithms. Therefore, standardization of acquisition procedures and cross-device validation are essential to ensure reproducibility and facilitate clinical translation. Additionally, integrating OCTA with OCT and VEP in future studies will provide a more comprehensive understanding of retinal and visual pathway changes in MS.

### 4.8. Limitations of the Study

Our study has several limitations. First, OCTA imaging is susceptible to artifacts—such as motion, segmentation errors, and media opacities—that can affect measurement accuracy. Patients with a history of ON may have poorer fixation, increasing artifact risk. Furthermore, the clinical relevance of OCTA remains limited by sparse longitudinal data and potential confounding from vascular comorbidities. Rigorous quality control is essential for reliable data interpretation. In our study, OCTA image quality was assessed using the device Q-value, integrating signal intensity, motion, and focus parameters; scans below the Q-value threshold corresponding to ~25 dB were excluded. In total, only ~5% of OCTA images were excluded due to artifacts, reflecting rigorous quality control and use of modern OCTA technology, which likely reduced measurement error compared to some earlier studies. Second, the cross-sectional design precludes causal inferences, and longitudinal studies are needed to determine whether retinal vascular parameters can predict disease progression. Third, correlations with structural OCT and electrophysiological measures such as VEP were not assessed in this study; these analyses are planned for future research to explore relationships between retinal microvascular, structural, and functional changes in MS. Fourth, our cohort did not include patients with progressive MS, limiting generalizability to this subgroup. This study included only patients with relapsing forms of MS; therefore, findings may not apply to primary progressive MS, where age-related and vascular comorbidities could differently affect OCTA parameters. Fifth, age influences retinal vessel densities, and our sample’s age distribution may confound some associations; future studies should stratify by age or include age-matched controls. Sixth, selection bias might have occurred, as patients were recruited during routine visits, possibly underrepresenting individuals with severe disability or other comorbidities. Seventh, OCTA measurements are device-specific, which hampers direct comparison across different platforms; standardization is necessary before clinical translation. All OCTA parameters analyzed in this study were automatically generated by the device’s AngioAnalytics software, which ensures standardization but may limit manual adjustment for individual segmentation errors. Eight, current OCTA metrics do not differentiate between vessel constriction, shrinkage, or true vessel loss, complicating interpretation regarding underlying pathology.

Finally, systemic comorbidities such as hypertension or diabetes can affect retinal vasculature independently of MS [35,36]. Although uncontrolled cases were excluded, their potential influence cannot be completely ruled out. Future studies should therefore systematically analyze the impact of systemic comorbidities and integrate OCTA with OCT and VEP assessments to comprehensively evaluate retinal and visual pathway changes in MS.

## 5. Conclusions

Our findings support the concept that retinal microvascular alterations, as measured by OCTA, are associated with clinical and radiological markers of disease activity and neurodegeneration in MS. The correlations with brain atrophy and disability suggest that OCTA could serve as a non-invasive biomarker for disease monitoring. Notably, SVP and DVP vessel densities demonstrated predictive value for achieving NEDA-3 status, emphasizing their potential in clinical practice. However, the cross-sectional design of this study limits causal interpretation, and future longitudinal studies are essential to validate these associations, assess the predictive value of OCTA parameters, and determine their utility in monitoring disease progression and response to therapy. Future longitudinal studies are essential to establish whether OCTA parameters can predict disease progression and response to therapy. Standardization of imaging protocols and validation across platforms are critical steps toward integrating OCTA into routine MS management. Additionally, mechanistic studies exploring the causal role of vascular changes could unveil novel therapeutic targets aimed at neuroprotection and vascular health in MS.

## Figures and Tables

**Table 1:** Demographic and clinical characteristics of patients and healthy controls.

	HCs	RMS Total	MS-ON	MS-nON	MS-ON vs. MS-nON *p* Value	MS-ON vs. HCs *p* Value	MS-nON vs. HCs *p* Value
Participants, *n* Female, n (%)	37 23 (62)	117 74 (63)					
Eyes	74	234	89 (38%)	145 (62%)			
Demographic characteristics							
Age at FUV, mean (SD), years	38.9 (12.9)	38 (10.4)	36.9 (10.4)	38.5 (10.4)	NS	NS	NS
Clinical characteristics							
Disease duration at FUV, median (IQR), years	–	8 (4–13)	11 (5.5–14)	6.5 (3–12)	0.001	–	–
Time from the last relapse to FUV, years, median IQR	–	1.5 (0.5–3.5)	2 (0.5–4)	1.2 (3–12)	NS	–	–
EDSS score at FUV, median (IQR)	–	2.5 (2–3.5)	2.5 (2–3.5)	2.5 (2–3.5)	NS	–	–
OCTA measures							
SVP, median (IQR), %	49.3 (47.8–50.1)	49.3 (48.3–50.2)	48.9 (47.9–50.1)	49.5 (48.5–50.4)	0.024	0.049	NS
DVP, median (IQR), %	52.7 (52.7–54.8)	54.2 (53.2–54.7)	54.1 (53.9–54.5)	54.2 (53.2–54.8)	NS	NS	NS
FAZ area, median (IQR), mm^2^	0.24 (0.19–0.29)	0.23 (0.17–0.3)	0.26 (0.19–0.3)	0.23 (0.15–0.29)	NS	NS	NS
FAZ FD, median (IQR), %	51.2 (49–54)	51.6 (49.1–53.7)	51.9 (48.9–54.2)	51.6 (49.1–53.6)	NS	NS	NS
RPC VD, median (IQR), %	49.4 (47.9–51)	48 (45.9–49.7	46.1 (42.9–48.5)	48.7 (47.2–50)	0.001	0.001	0.011
MRI measures at FUV							
FLAIR lesions volume (mL), median (IQR)	–	4.2 (2.3–8.9)	3.3 (2.4–11)	4.3 (2.3–8)	NS	–	–
T1 hypointense lesions volume (mL), median (IQR)	–	2.2 (1.1–4.6)	2.2 (1.1–4.6)	1.9 (1–5.6)	NS	–	–
WB volume (mL), median (IQR)	–	1544 (1507–1588)	1544 (1492–1582)	1545 (1511–1588)	NS	–	–
WB volume normative percentile, median (IQR)	–	11.9 (3.8–46)	8 (2.3–46.8)	17 (4.4–46)	NS	–	–
GM volume (mL), median (IQR)	–	919 (886–944)	912 (885–953)	923 (886–943)	NS	–	–
GM volume normative percentile, median (IQR)	–	16 (1.6–44)	9.8 (1–42)	17 (2–52)	NS	–	–

**Table 2:** Correlation analysis.

	OCTA Measures				
Variables	SVP (%)	DVP (%)	FAZ Area (mm^2^)	FAZ FD (%)	RPC VD (%)
Age (years)	−0.16 *	−0.12	0.11	−0.21 **	0.09
Last relapse period (years)	−0.25 **	−0.18 **	−0.04	−0.21 **	−0.19 **
Disease duration (years)	−0.19 **	−0.07	0.05	−0.14 *	−0.22 **
EDSS	0.04	−0.08	0.14 *	−0.05	0.01
FLAIR lesions volume (mL)	−0.07	0.05	−0.01	−0.10	−0.22 **
FLAIR new lesions volume (mL)	0.26 **	0.23 *	0.04	0.06	−0.07
T1-hypointense lesions volume (mL)	0.10	0.08	0.19 *	−0.06	0.11
WB volume (mL)	0.19 *	0.16 *	−0.05	−0.03	0.27 **
GM volume (mL)	0.19 *	0.17 *	−0.12	−0.05	0.16 *

*—Correlation is significant at the 0.05 level (2-tailed), **—Correlation is significant at the 0.01 level (2-tailed).

## Data Availability

The data presented in this study are available upon request from the corresponding author.

## Abbreviations

The following abbreviations are used in this manuscript:

AUC	Area under the curve
CI	Confidence Interval
DMT	Disease Modifying Therapy
DVP	Deep vascular plexus
EDSS	Expanded Disability Status Scale
FAZ	Foveal avascular zone
FAZ FD	Foveal avascular zone floor density
FLAIR	Fluid-attenuated inversion recovery
FUV	follow-up visit
Gd	gadolinium
GM	Gray Matter
HCs	Healthy Controls
IQR	Interquartile Range
MRI	Magnetic Resonance Imaging
NEDA	No Evidence of Disease Activity
OCT	Optical coherence tomography
OCTA	Optical coherence tomography angiography
ON	Optic neuritis
RMS	Relapsing Multiple Sclerosis
PwMS	Patients with Multiple sclerosis
ROC	Receiver operating characteristics
RPC	Radial peripapillary capillary
SD	Standard deviation
SVP	Superficial vascular plexus
VD	Vessel density
WB	Whole Brain
